# Psychological capital and death anxiety in pancreatic cancer patients: a latent profile analysis

**DOI:** 10.3389/fpsyt.2025.1627422

**Published:** 2025-09-10

**Authors:** Dajun Yang, Tianyu She, Gui Gui, Linwei Li, Zhiwen Zhou, Lu Liu, Nian Liu

**Affiliations:** ^1^ Department of Radiology, Affiliated Hospital of North Sichuan Medical College, Nanchong, China; ^2^ Key Laboratory of Digital-Intelligent Disease Surveillance and Health Governance, North Sichuan Medical College, Nanchong, Sichuan, China; ^3^ Department of Medical Imaging Function, Xi’an Electric Power Central Hospital, Xi’an, Shaanxi, China; ^4^ School of Medical Imaging, North Sichuan Medical College, Nanchong, China; ^5^ Mental Health Center, Affiliated Hospital of North Sichuan Medical College, Nanchong, China

**Keywords:** pancreatic cancer, psychological capital, death anxiety, perceived stress, social support, latent profile analysis

## Abstract

Prior research has predominantly adopted variable-centered approaches to demonstrate significant correlations between psychological capital and death anxiety. However, few studies have investigated the heterogeneity of these constructs among patients with pancreatic cancer. To address this gap, we employed a random sampling method to recruit 513 patients with pancreatic cancer. Latent profile analysis was conducted to examine their psychological capital and death anxiety profiles, followed by univariate analysis and multinomial logistic regression to identify influencing factors. The results revealed three distinct profiles: high psychological capital–low death anxiety, moderate psychological capital–moderate death anxiety, and low psychological capital–high death anxiety. Key determinants included Gender, age, place of residence, and cancer stage. These findings enhance our understanding of the psychological recovery trajectory in pancreatic cancer patients, enabling clinicians to develop targeted interventions based on distinct psychological profiles to improve mental health outcomes.

## 1 Introduction

Pancreatic cancer is one of the malignant tumors with the lowest five-year survival rates, which is less than 10% (1, 2). It ranks as the seventh leading cause of cancer-related mortality worldwide (3), claiming nearly half a million lives annually (4), with particularly high mortality rates in developed countries such as the United States (5, 6). Despite advancements in multidisciplinary treatments—including surgical intervention, chemotherapy, and nutritional support (7, 8)—the disease’s insidious progression and limited therapeutic efficacy continue to inflict severe physical and psychological suffering (9). Notably, over 30% of patients exhibit clinically significant death anxiety at diagnosis (10), while more than 50% experience depressive tendencies during treatment (11). Death anxiety refers to the fear, worry, or unease of patients about death-related events of themselves or others (12). Death anxiety not only includes specific manifestations such as fear of the death process (13), worry about the unknown after death (14), and anxiety about the loss of the meaning of life (15), but also may be accompanied by physiological manifestations such as palpitations (16), asphyxia (17), and cognitive avoidance (18). Previous studies have found that death anxiety can exacerbate depression (19), sleep disorders (20), social withdrawal (21), and treatment termination in cancer patients (22). For example, in the study by Gui et al. (23), it was explored that family support could effectively alleviate the death anxiety of breast cancer patients. However, few studies have paid attention to the death anxiety of pancreatic cancer patients. This neglect is deplorable. According to data released by the National Cancer Center in 2024, the 5-year survival rate of pancreatic cancer in China is only 7.2% (24). Compared with other adenocarcinomas or cancers, with effective treatment at early detection, the 5-year survival rate is relatively high, and patients have a longer survival expectancy (25–27). On the other hand, the research of pancreatic cancer focuses more on treatment methods and pathological mechanisms (28, 29), while the research on the psychological level of death anxiety of pancreatic cancer patients is relatively few, and there is a lack of effective intervention measures. Therefore, by studying the heterogeneity of death anxiety in patients with pancreatic cancer, this study developed measures for different categories of pancreatic cancer patients to help them reduce death anxiety, improve their quality of life, and prolong their survival.

In recent years, a large number of researchers have begun to pay attention to the correlation between psychological capital and death anxiety (30–32). Psychological Capital refers to an individual’s positive psychological resources, including resilience, self-efficacy, and adaptive coping mechanisms (33, 34), and is a critical determinant of treatment outcomes in pancreatic cancer (35). Characterized by its high lethality, rapid physical deterioration, treatment-related toxicities, and relatively low overall survival rates (36–38), this disease context may be mitigated by psychological capital, which buffers psychological trauma and fosters treatment resilience. Preliminary evidence suggests that enhanced psychological resources in pancreatic cancer patients often correlate with better treatment adherence (39, 40). Specifically, patients with higher psychological capital demonstrate greater tolerance for aggressive treatment regimens, reduced engagement in health-damaging behaviors, and improved tolerance during completion of neoadjuvant therapy (41, 42). However, previous studies have never explored the relationship between psychological capital and death anxiety in patients with pancreatic cancer, and even fewer studies have analyzed the heterogeneity of psychological capital and death anxiety in patients with pancreatic cancer and their influencing factors.

Recent cross-sectional studies have gradually elucidated the complex relationship between psychological capital and death anxiety in pancreatic cancer patients (35, 43, 44). However, these studies exhibit significant limitations in methodology and theoretical depth. For instance, while prior research highlights negative correlations between death anxiety and dimensions of psychological capital—such as hope, self-efficacy, resilience, and optimism (45, 46)—the underlying mechanisms remain underexplored. Specifically, hope has been shown to buffer death anxiety through goal-directed thinking and positive attribution patterns, enabling patients to reinterpret disease outcomes (47, 48). Optimism mitigates catastrophic cognitions by framing mortality as a universal human experience (49). In terms of emotional regulation, self-efficacy enhances perceived control over treatment processes (50, 51), while resilience reduces emotional exhaustion by fostering acceptance of disease progression (52). For example, Marinelli et al. (53) found that advanced pancreatic cancer patients with high self-efficacy reported significantly lower death anxiety scores than those with low self-efficacy. Collectively, these findings suggest that robust psychological capital confers resilience against death anxiety (54). From a neurobiological perspective, psychological capital interventions have been linked to strengthened prefrontal cortex regulation of the limbic system, inhibition of amygdala hyperactivation, and reduced cortisol levels (55–57). Such neuroendocrine modulation disrupts the fear-stress-anxiety cycle, underscoring the therapeutic potential of psychological capital in clinical settings.

However, some scholars argue that death anxiety can also undermine psychological capital (58, 59). Death anxiety triggers persistent worry and rumination, diverting cognitive resources toward imagined mortality scenarios (60, 61). This state undermines individuals’ ability to cope with real-world challenges, eroding self-efficacy (62, 63). From an existential perspective, death anxiety may provoke profound angst about the meaning of life (64–66). Specifically, when individuals succumb to self-denigration over perceived wasted potential, the hope dimension of psychological capital is directly impaired, leaving them unable to sustain goal-directed optimism.

The inconsistent findings regarding the relationship between death anxiety and psychological capital may partially stem from limitations in research perspectives. Previous studies have predominantly employed variable-centered approaches to examine associations between psychological constructs, yet this methodology often overlooks individual heterogeneity. Pancreatic cancer patients may exhibit distinct patterns of psychological capital and death anxiety based on their unique experiences, coping strategies, and disease trajectories (13, 34). Neglecting this heterogeneity risks overgeneralized conclusions and constrains the development of targeted interventions.

Furthermore, research investigating influential factors of psychological capital and death anxiety in pancreatic cancer patients remains relatively scarce. Existing studies have primarily focused on demographic characteristics, presenting a unidimensional perspective (45, 67). Perceived stress, defined as an individual’s subjective cognitive appraisal of stressors, emphasizes personal interpretation of challenging events (68, 69). Pancreatic cancer patients frequently endure intense psychological stress, which may undermine self-efficacy by fostering doubts about recovery capabilities (70, 71). Concurrently, heightened stress levels may induce physiological symptoms such as palpitations and insomnia, potentially misinterpreted as harbingers of imminent mortality, thereby exacerbating death anxiety (72, 73). Beyond perceived stress, social support emerges as another potential determinant, encompassing material assistance, emotional comfort, information sharing, and sense of belonging within social networks (74, 75). Prognostic guidance and rehabilitation protocols from specialized medical teams can effectively correct patients’ cognitive biases regarding mortality (76, 77). Nevertheless, previous research has rarely explored how perceived stress and social support influence psychological capital and death anxiety in this population.

This study therefore addresses two primary questions: 1) What distinct subgroups exist regarding psychological capital and death anxiety among pancreatic cancer patients? 2) Which factors influence these psychological constructs across different subgroups? Latent Profile Analysis (LPA), a person-centered statistical approach, provides a robust framework for identifying subgroups with similar psychological characteristics (78). By applying LPA to pancreatic cancer patients, this research reveals distinct psychological capital and death anxiety profiles while exploring each subgroup’s defining attributes. Subsequently, we examine how demographic characteristics, perceived stress, and social support differentially impact these psychological constructs across identified latent classes. This dual approach not only enhances our understanding of psychological heterogeneity but also establishes foundations for personalized interventions. Healthcare professionals conducting psychological counseling should consider patients’ distinct psychological profiles while emphasizing individualized management of perceived stress and social support systems.

## 2 Method

### 2.1 Sample sources

This study aimed to use Latent Profile Analysis (LPA) to investigate the heterogeneity of psychological capital and death anxiety among pancreatic cancer patients. The Ethics Committee of North Sichuan Medical College approved the study. Data collection was conducted in several Grade A tertiary hospitals in Nanchong City and Xi’an City. Prior to data collection, all participants were informed of the study’s purpose, procedures, potential risks, benefits, and the voluntary nature of participation. Following the obtainment of informed consent, participants completed either paper or electronic questionnaires. Given the relatively long nature of the questionnaire, completion of all items required approximately 8 minutes.

Inclusion Criteria. (1) Patients were diagnosed with pancreatic ductal adenocarcinoma through pathological or imaging examinations and staged as I-IV according to the AJCC 8th Edition staging system (79). (2) Patients were aged over 18, had no significant cognitive impairment, and were capable of signing informed consent independently. (3) Patients had an expected survival period of at least 3 months, as assessed by tumor oncologists. (4) Patients were native Chinese speakers with no significant language barriers or reading difficulties.

Exclusion Criteria. (1) Patients with severe cognitive impairments, such as dementia, delirium, or active mental illnesses. (2) Patients who had undergone systematic psychological intervention or adjustment of psychotropic medications within the past 4 weeks. (3) Patients requiring long-term bed rest or completely lacking self-care ability. (4) Patients who had participated in other clinical trials involving psychological assessment within the past 3 months.

Data collection took place between June 2024 and December 2024. A total of 550 questionnaires were distributed, with 526 returned. During data cleaning, 13 questionnaires were excluded due to short time to fill (N=3), incomplete questionnaire filling (N=6), and too consistent responses (N=4). Given that 6 points of incomplete data had been excluded, there were no missing data at the item level in the remaining 513 questionnaires, and the list deletion method was used in descriptive and correlation analyses, which is recommended for low missing rates to minimize bias (80). For LPA and regression models, under the missing at random assumption, full information maximum likelihood estimation was used to account for any residual missing, ensuring unbiased parameter estimates (81). This resulted in 513 valid questionnaires, yielding an effective response rate of 93.27%. The sample included 256 male and 257 female patients. Detailed demographic information is provided in **Table 1**.

**Table 1:** Sample demographic information.

Variables	Items	Numbers (N)	Frequency (%)
Gender	Male	256	49.9%
	Female	257	50.1%
Age	18–30 years old	61	11.9%
	31–45 years old	59	11.5%
	46–60 years old	243	45.6%
	Above 61 years old	159	31%
Educational Background	Primary school	107	20.9%
	Junior high school	116	22.6%
	Senior high school	118	23%
	Junior college	114	22.2%
	Undergraduate	37	7.2%
	Master’s degree	15	2.9%
	Doctoral degree	6	1.2%
Residential Area	Rural	239	46.6%
	Urban	274	53.4%
Marital Status	Married	403	78.6%
	Single	54	10.5%
	Divorced	39	7.6%
	Widowed	17	7.3%
Cancer Stage	I	73	14.2%
	II	243	47.4%
	III	106	20.7%
	IV	91	17.7%

### 2.2 Measurement tools

#### 2.2.1 Psychological Capital Scale

The Psychological Capital Scale used in this study was adapted from Zhang (82), consisting of 26 items across four dimensions: self-efficacy, resilience, hope, and optimism. This scale has been widely used among Chinese populations (83, 84) and has demonstrated strong cultural adaptability and reliability. For instance, Zhou et al. (85) employed this scale to measure psychological capital among Chinese nurses. The 26-item version was selected to assess psychological capital in patients with pancreatic cancer. Responses were collected using a 7-point Likert scale (1=“strongly disagree,” 7=“strongly agree”), with higher scores indicating more substantial psychological capital. The Cronbach’s α coefficient for this scale in the current study was 0.952.

#### 2.2.2 Death Anxiety Scale

The Death Anxiety Scale was adapted from Templer (86), comprising 15 items across four dimensions: affective (6 items), stress and suffering (4 items), time awareness (2 items), and cognitive (3 items). This scale has been widely applied to measure death anxiety among Chinese cancer populations (67, 87) and was translated into Chinese and validated for cultural adaptability and reliability by Che et al. (88). All 15 items were used to assess death anxiety in pancreatic cancer patients. Responses were collected using a 7-point Likert scale (1=“strongly disagree,” 7=“strongly agree”), with higher scores indicating greater death anxiety. The Cronbach’s α coefficient for this scale was 0.882.

#### 2.2.3 Social Support Scale

The Social Support Scale was adapted from Zimet et al. (89), consisting of 12 items across three dimensions: support from others, friends, and family. This scale has been widely used to measure social support among Chinese populations (120, 121) and has been translated into Chinese, validated for cultural adaptability, and tested for reliability by Yang et al. (90). All 12 items were used to assess social support in patients with pancreatic cancer. Responses were collected using a 7-point Likert scale (1=“strongly disagree,” 7=“strongly agree”), with higher scores indicating greater social support. The Cronbach’s α coefficient for this scale was 0.75.

#### 2.2.4 Perceived Stress Scale

The Perceived Stress Scale was adapted from Cohen et al. (91), comprising 14 items. This scale was translated into Chinese, validated for cultural adaptability, and tested for reliability by Yang and Huang (92). All 14 items were used to assess perceived stress among pancreatic cancer patients. Responses were collected using a 7-point Likert scale (1=“strongly disagree,” 7=“strongly agree”), with higher scores indicating greater perceived stress. The Cronbach’s α coefficient for this scale was 0.857.

## 3 Result

### 3.1 Common method bias test

Following the approach used by Podsakoff et al. (93), we employed an anonymous data collection method to gather self-reported data from participants, thereby reducing potential biases in the responses. Subsequently, we utilized Harman’s single-factor test to examine common method bias. The results indicated that, without rotation, a total of 12 factors with eigenvalues greater than one were extracted. The first factor explained 24.114% of the variance, which did not exceed the critical threshold of 40%. This suggests that the study is free from issues related to common method bias.

### 3.2 Descriptive statistics and correlation analysis

We conducted descriptive statistics and correlation analyses on psychological capital, death anxiety, social support, perceived stress, and demographic information among pancreatic cancer patients, as shown in **Table 2**. The results revealed the following significant correlations: psychological capital was negatively correlated with death anxiety (r=-0.491, P < 0.001) and perceived stress (r=-0.224, P < 0.001), and positively correlated with social support (r=0.429, P < 0.001). Death anxiety was negatively correlated with social support (r=-0.412, P < 0.001) and positively correlated with perceived stress (r=0.252, P < 0.001). Social support was negatively correlated with perceived stress (r=-0.441, P < 0.001). From a demographic perspective, gender was significantly correlated with psychological capital, death anxiety, perceived stress, and social support. Residence was also significantly correlated with psychological capital, death anxiety, perceived stress, and social support. Cancer stage was significantly correlated with psychological capital and death anxiety.

**Table 2:** Descriptive statistics of demographic information of variables.

Variables	M	SD	1	2	3	4	5	6	7	8	9	10
1. Psychological Capital	4.112	1.286	1									
2. Death Anxiety	3.222	0.825	-0.491**	1								
3. Perceived Stress	2.14	0.918	-0.224**	0.252**	1							
4. Social Support	4.010	1.014	0.429**	-0.412**	-0.441**	1						
5. Gender	-	-	0.290**	-0.247**	-0.185**	0.178**	1					
6. Age	-	-	0.337**	-0.448**	-0.165**	0.262**	0.189**	1				
7. Educational Background	-	-	0.037	-0.077	0.041	-0.026	-0.026	0.009	1			
8. Place of Residence	-	-	0.128**	-0.116**	-0.101*	0.138**	0.068	0.067	0.028	1		
9. Marital Status	-	-	-0.049	0.068	0.034	-0.038	-0.024	-0.041	0.009	-0.029	1	
10. Cancer Stage	-	-	-0.117**	0.058*	-0.093	-0.033	-0.024	-0.006	0.036	-0.02	-0.07	1

*p<0.05; **p<0.01; ***p<0.001.

### 3.3 Latent profile analysis

This study employed the LPA method to evaluate potential models with 1 to 5 latent profiles, aiming to determine the optimal fitting model, as detailed in **Table 3**. Analyses were conducted using Mplus version 8.3 (94) with robust maximum likelihood estimation to handle potential non-normality and provide bias-corrected standard errors. Models were estimated using 500 random starts and 100 final-stage optimizations to ensure convergence on global maxima. As the number of profiles increased, the information criteria AIC, BIC, and aBIC consistently decreased, indicating improved model fit. Furthermore, the Lo-Mendell-Rubin likelihood ratio test (LMRT) and the bootstrapped likelihood ratio test (BLRT) values remained significant. However, upon further comparison, we found that the Entropy value for the 3-profile model was significantly higher than those for the 2-, 4-, and 5-profile models. Entropy is primarily used to assess the accuracy of latent class assignment in a model, with values closer to 1 indicating better classification quality. Therefore, the 3-profile model was deemed more reasonable.

**Table 3:** Potential profile fitting analysis of psychological capital and death anxiety.

Profile	AIC	BIC	aBIC	Entropy	LMR (P)	BLRT (P)	Smallest proportion per class
1	33968.990	34130.130	34009.511				
2	32076.734	32322.670	32138.568	0.915	<0.001	<0.001	0.298/0.701
3	31319.688	31650.429	31402.844	0.967	<0.001	<0.001	0.172/0.643/0.185
4	30792.368	31207.915	30896.847	0.912	0.012	<0.001	0.351/0.156/0.318/0.175
5	30559.227	31059.579	30685.028	0.927	0.0006	<0.001	0.156/0.037/0.148/0.366/0.292

### 3.4 Subgroup classification

**Figure 1:**
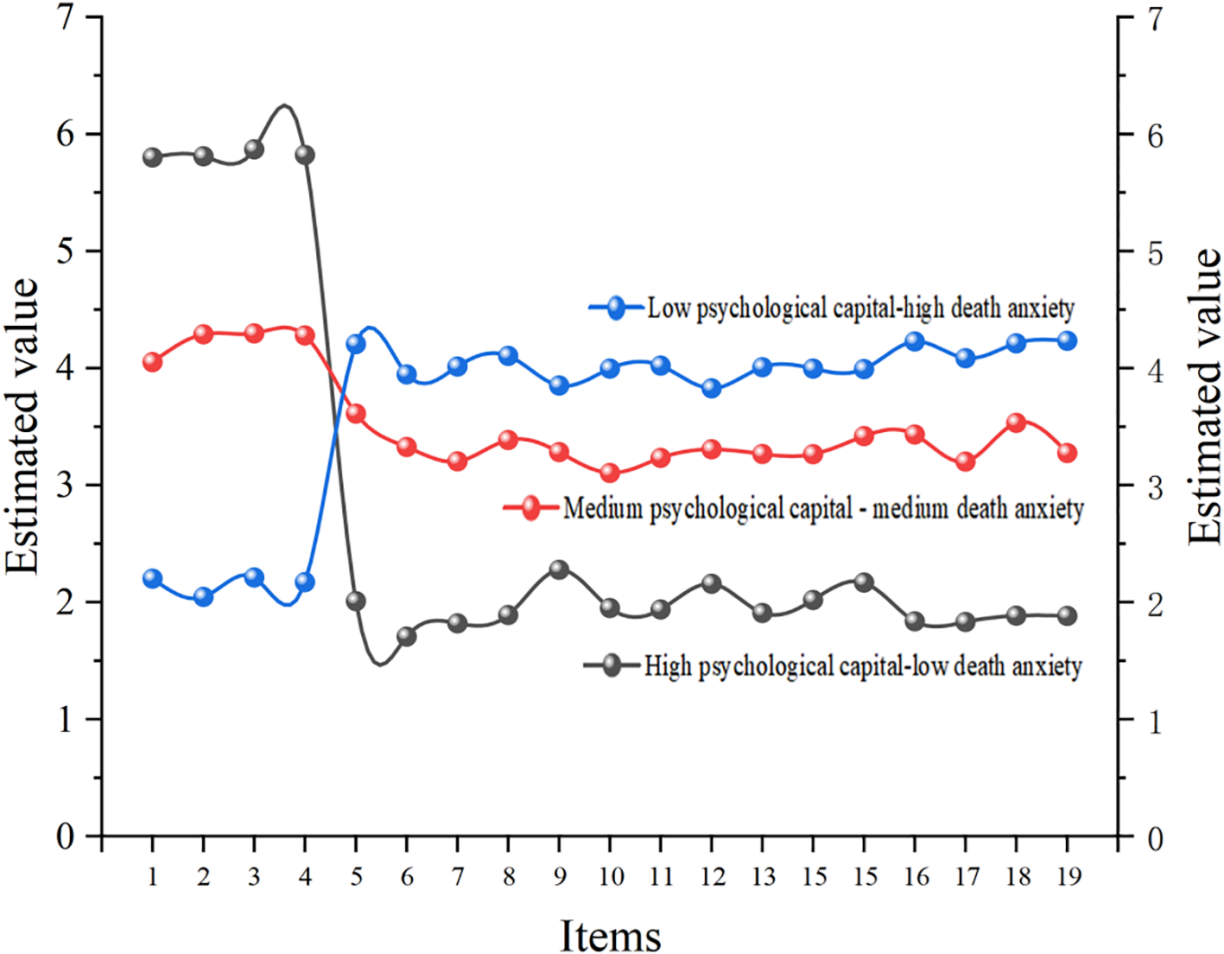
Potential profile results of psychological capital and death anxiety. The 1–4 items are the four dimensions of psychological capital, namely self-efficacy, resilience, hope, and optimism. The 5–19 items were death anxiety.

The latent profile analysis identified three subgroups, as illustrated in **Figure 1**. The first subgroup was labeled as “high psychological capital-low death anxiety,” accounting for 17.15% of the sample. The second subgroup was labeled as “moderate psychological capital-moderate death anxiety,” representing 64.33% of the sample. The third subgroup was named “low psychological capital-high death anxiety,” comprising 18.52% of the sample. This indicates that most pancreatic cancer patients exhibit some degree of death anxiety and psychological capital.

### 3.5 Single-factor analysis of psychological capital and death anxiety subgroups

We compared the three psychological capital and death anxiety subgroups in terms of demographic characteristics, social support, and perceived stress using single-factor analysis. The results demonstrated significant differences in psychological capital and death anxiety across gender, age, residence, and cancer stage (P < 0.001). In contrast, no significant differences were found for education (P=0.208) or marital status (P=0.250). Further details are provided in **Table 4**.

**Table 4:** Univariate analysis of latent profiles of psychological capital and death anxiety.

Variable	Items	High psychological capital - low death anxiety (n=88)	Moderate psychological capital-moderate death anxiety (n=330)	Low psychological capital-high death anxiety (n=95)	χ ²	P
Gender	Male	23 (9.0%)	154 (60.15%)	79 (30.85%)	63.289	<0.001
	Female	65 (25.29%)	176 (68.48%)	16 (6.23%)		
Age	18–30 years old	3 (4.92%)	26 (42.62%)	32 (52.46%)	107.754	<0.001
	31–45 years old	3 (5.08%)	34 (57.63%)	22 (37.29%)		
	46–60 years old	31 (13.25%)	170 (72.65%)	33 (14.10%)		
	Above 61 years old	51 (32.08%)	100 (62.89%)	8 (5.03%)		
Educational Background	Primary school	18 (16.82%)	68 (63.55%)	21 (19.63%)	15.655	0.208
	Junior secondary school	18 (15.52%)	73 (62.93%)	25 (21.55%)		
	Senior secondary school	22 (18.64%)	79 (66.95%)	17 (14.41%)		
	College	15 (13.16%)	76 (66.67%)	23 (20.17%)		
	Undergraduate	9 (19.15%)	22 (46.81%)	16 (34.04%)		
	Master’s degree	2 (13.33%)	10 (66.67%)	3 (20.00%)		
	Doctoral degree	4 (66.67%)	2 (33.33%)	0		
Residential Area	Rural	31 (12.97%)	148 (61.93%)	60 (25.10%)	15.448	<0.001
	Urban	57 (20.80%)	182 (66.42%)	35 (12.78%)		
Marital Status	Married	71 (17.62%)	261 (64.76%)	71 (17.62%)	7.839	0.250
	Single	7 (12.96%)	39 (72.22%)	8 (14.82%)		
	Divorced	7 (17.95%)	19 (48.72%)	13 (33.33%)		
	Widowed	3 (17.65%)	11 (64.7%)	3 (17.65%)		
Cancer Stage	I	20 (27.40%)	35 (47.95%)	18 (24.66%)	60.996	<0.001
	II	33 (13.58%)	194 (79.84%)	16 (6.58%)		
	III	18 (16.98%)	57 (53.77%)	31 (29.25%)		
	IV	17 (18.68%)	44 (48.35%)	30 (32.97%)		

### 3.6 Logistic regression analysis of factors influencing the latent categories of psychological capital and death anxiety

We treated the latent categories of psychological capital and death anxiety in pancreatic cancer patients as the dependent variables, using the variables with statistical significance from the single-factor analysis as independent variables. The “low psychological capital-high death anxiety” group was used as the reference category. Prior to regression, multicollinearity was assessed using Variance Inflation Factors (VIF), with all values ranging from 1.017 to 1.329 (mean VIF=1.137), well below the threshold of 5, indicating no multicollinearity issues (95). Unordered multinomial logistic regression analysis was conducted to examine the relationships.

Comparison of the high psychological capital-low death anxiety group with the reference group. The probability of males belonging to this group was significantly lower than that of females (OR=0.073, 95% CI=[0.030, 0.182], P < 0.001), suggesting that females are more likely to maintain high psychological capital and alleviate death anxiety. The probabilities of belonging to this group were significantly lower for the 18 - 30 (OR=0.011, P < 0.001), 31 - 45 (OR=0.053, P < 0.001), and 46 - 60 (OR=0.195, P=0.002) age groups compared to the above 61 years old age group, indicating that younger individuals exhibited lower psychological capital and higher death anxiety, possibly related to life stress or health risk perception. Rural residents had a significantly lower probability of belonging to this group compared to urban residents (OR=0.44, 95% CI=[0.196, 0.986], P=0.046). Stage II patients were significantly more likely to belong to this group than early-stage patients (OR=8.27, 95% CI=[2.56, 26.713], P < 0.001). Each unit increase in social support significantly increased the likelihood of belonging to this group by 6.6-fold (OR=6.602, P < 0.001), highlighting the reinforcing effect of social support on psychological capital.

Comparison of the moderate psychological capital-moderate death anxiety group with the reference group: The probability of males belonging to this group was also significantly lower than that of females (OR=0.18, 95% CI=[0.086, 0.378], P < 0.001), showing consistent gender differences across both groups. The probabilities for the 18 - 30 (OR=0.053, P < 0.001) and 31 - 45 (OR=0.172, P=0.002) age groups were significantly lower than that of the older age group, while the 46–60 age group showed no significant difference (P=0.106). Stage II patients were 10.07 times more likely to belong to this group than early-stage patients (OR=10.07, 95% CI=[4.089, 24.801], P < 0.001), indicating that disease progression exacerbates the co-occurrence of psychological capital and death anxiety. Increased social support significantly enhanced the likelihood of belonging to this group (OR=2.722, 95% CI=[1.893, 3.915], P < 0.001). However, this effect was weaker compared to the “high psychological capital-low death anxiety” group. Further details are provided in **Tables 5**–**10**.

**Table 5:** Logistic regression analysis of factors influencing the latent categories of psychological capital and death anxiety (age).

Classification	Variables	Items	Regression coefficient	Standard error	Wald χ²	P	OR	95% CI
High psychological capital - Low death anxiety	Age	18–30 years old	-4.55	0.846	28.922	<0.001	0.011	[0.002,0.055]
		31–45 years old	-2.941	0.835	12.392	<0.001	0.053	[0.01,0.272]
		46–60 years old	-1.634	0.535	9.339	0.002	0.195	[0.068,0.556]
		Above 61 years old (refer)						
Medium psychological capital - Medium death anxiety	Age	18–30 years old	-2.94	0.558	27.745	<0.001	0.053	[0.018,0.158]
		31–45 years old	-1.76	0.574	9.413	0.002	0.172	[0.056,0.53]
		46–60 years old	-0.764	0.472	2.618	0.106	0.466	[0.185,1.175]
		Above 61 years old (refer)						

**Table 6:** Logistic regression analysis of factors influencing the latent categories of psychological capital and death anxiety (gender).

Classification	Variables	Items	Regression coefficient	Standard error	Wald χ²	P	OR	95% CI
High psychological capital - Low death anxiety	Gender	Male	-2.611	0.463	31.834	<0.001	0.073	[0.03,0.182]
		Female(refer)						
Medium psychological capital - Medium death anxiety	Gender	Male	-1.713	0.377	20.62	<0.001	0.18	[0.086, 0.378]
		Female(refer)						

**Table 7:** Logistic regression analysis of factors influencing the latent categories of psychological capital and death anxiety (residential area).

Classification	Variable	Items	Regression coefficient	Standard error	Wald χ²	P	OR	95% CI
High psychological capital - Low death anxiety	Residential Area	Rural	-0.82	0.412	3.973	0.046	0.44	[0.196,0.986]
		Urban(refer)						
Medium psychological capital - Medium death anxiety	Residential Area	Rural	-0.447	0.324	1.905	0.168	0.64	[0.339,1.206]
		Urban(refer)						

**Table 8:** Logistic regression analysis of factors influencing the latent categories of psychological capital and death anxiety (cancer stage).

Classification	Variables	Items	Regression coefficient	Standard error	Wald χ²	P	OR	95% CI
High psychological capital - Low death anxiety	Cancer stage	I	1.327	0.671	3.907	0.048	3.77	[1.011,14.054]
		II	2.113	0.598	12.47	<0.001	8.27	[2.56,26.713]
		III	0.12	0.598	0.04	0.841	1.128	[0.349,3.639]
		IV(refer)						
Medium psychological capital - Medium death anxiety	Cancer stage	I	0.472	0.517	0.832	0.362	1.603	[0.582,4.417]
		II	2.31	0.46	25.221	<0.001	10.07	[4.089,24.801]
		III	-0.089	0.431	0.042	0.837	0.915	[0.393,2.131]
		IV(refer)						

**Table 9:** Logistic regression analysis of factors influencing the latent categories of psychological capital and death anxiety (social support).

Classification	Variable	Regression coefficient	Standard error	Wald χ²	P	OR	95% CI
High psychological Capital - Low Death Anxiety	Social Support	1.887	0.255	54.951	<0.001	6.602	[4.008,10.874]
Medium psychological Capital - Medium Death Anxiety	Social Support	1.001	0.185	29.168	<0.001	2.722	[1.893,3.915]

**Table 10:** Logistic regression analysis of factors influencing the latent categories of psychological capital and death anxiety (perceived stress).

Classification	Variable	Regression coefficient	Standard error	Wald χ²	P	OR	95% CI
High psychological Capital - Low Death Anxiety	Perceived Stress	-0.138	0.245	0.319	0.572	0.871	[0.539,1.407]
Medium psychological Capital - Medium Death Anxiety	Perceived Stress	-0.027	0.184	0.022	0.883	0.973	[0.678,1.397]

## 4 Discussion

### 4.1 Latent profile analysis

This study employed the LPA method to investigate the heterogeneity of psychological capital and death anxiety among pancreatic cancer patients. Participants were categorized into three latent profiles: high psychological capital-low death anxiety, moderate psychological capital-moderate death anxiety, and low psychological capital-high death anxiety. The moderate psychological capital-moderate death anxiety group had the highest sample proportion. These findings partially align with previous studies. For instance, Teng et al. (96) identified similar classification patterns based on psychological factors in a study on nurses’ psychological capital. However, that study only focused on the heterogeneity of psychological capital and did not explore the distinct grouping characteristics of death anxiety among pancreatic cancer patients. The present study further advances the understanding of this specific patient population by providing detailed profiles of proportions and characteristics.

Based on social cognitive theory, psychological capital is viewed as an expression of individual behavior in different states (97). These states reflect an individual’s behavior at a specific moment and are a manifestation of their psychological capital. Psychological capital comprises elements such as self-efficacy, optimism, hope, and resilience. The varying combinations and levels of these elements result in diverse psychological states among patients when facing pancreatic cancer. Death anxiety, as a strong negative emotion, interacts with psychological capital. Patients with different traits exhibit varying dimensions of psychological capital when coping with the disease, leading to differences in the degree of death anxiety. These combinations of traits and states are manifested in real-life situations.

Among the three identified latent profiles, the “low psychological capital-high death anxiety” group represented a relatively small proportion. In contrast, the “moderate psychological capital-moderate death anxiety” group was significantly more representative. This suggests that the combination of moderate psychological capital and moderate death anxiety is a relatively common phenomenon in the pancreatic cancer patient population. Most patients do not possess extremely high psychological capital to cope with the disease effectively, nor do they have extremely low psychological capital, leading to complete despair. Instead, they hover between the two extremes, reflecting the complex psychological states of patients as they face the disease.

### 4.2 Analysis of influencing factors for different potential profiles

#### 4.2.1 Gender

This study found that gender is a core variable distinguishing the combination of states of psychological capital and death anxiety. In the “high psychological capital-low death anxiety” group, males had a significantly lower probability of belonging to this group compared to females. This result is consistent with previous research suggesting that females tend to have higher psychological resilience than males (98, 99). Social role theory posits that women tend to assume more emotional support roles in both family and society, potentially accumulating psychological resources through more frequent emotional expression and social interactions (100). Additionally, neuroendocrinological studies suggest that estrogen’s regulatory role in stress responses may enhance women’s ability to buffer death anxiety (101). However, this advantage may come at a cost, as women’s heightened vigilance to health threats may increase the risk of death anxiety (102). Nevertheless, the present study found that women still exhibited higher psychological capital, suggesting that they effectively transformed potential anxiety through social support networks.

Notably, gender differences remained significant in the “moderate psychological capital-moderate death anxiety” group, albeit with a reduced effect size, indicating that the protective effect of gender on psychological capital diminishes as death anxiety levels increase. When external pressures exceed the capacity of the social support system, the influence of gender differences on psychological resources may weaken (103).

#### 4.2.2 Age

Significant nonlinear trends characterized the relationship between age and psychological capital-death anxiety. The probability of 18 - 30-year-olds belonging to the “high psychological capital-low death anxiety” group was only 0.011 of that of older individuals. From a developmental psychology perspective, young adults face multiple transitional pressures, such as career orientation and economic independence, which may deplete short-term psychological capital (104). Additionally, the U-shaped curve theory of death anxiety suggests that young individuals’ conceptualization of death is less mature, and sudden health events (e.g., a cancer diagnosis) may trigger existential fear (105). In contrast, the 46–60 age group exhibited transitional characteristics, possibly reflecting the accumulation of psychological resources through life experiences, albeit still constrained by responsibilities such as childcare and care for the elderly. Notably, the oldest group (61 years and above) had the highest probability of belonging to the high psychological capital group, consistent with the paradox of aging theory. Older adults may alleviate anxiety by accepting death and reconstructing meaning (106). However, this advantage may be influenced by sample selection bias, as late-stage cancer patients are often older and may not have been fully included in the study.

#### 4.2.3 Residence

Residence significantly influenced the patterns of psychological capital and death anxiety. The probability of rural residents belonging to the “high psychological capital-low death anxiety” group was only 44% of that of urban residents, highlighting the profound impact of structural health inequalities. This may be attributed to the insufficient coverage of mental health services in rural areas, which is only one-third of that in urban areas (107), and the stigma that deters help-seeking behavior (108). Interestingly, urban-rural differences were not statistically significant in the “moderate psychological capital-moderate death anxiety” group, suggesting that structural factors may be buffered by individual resilience when psychological capital and anxiety are balanced.

#### 4.2.4 Cancer stage

The influence of cancer stage on psychological capital revealed significant phase-specific characteristics. The probability of Stage II patients belonging to the “high psychological capital-low death anxiety” group was 8.27 times higher than that of early-stage patients. According to post-traumatic growth theory, the initial shock of a disease diagnosis (Stage I) may disrupt psychological equilibrium. In contrast, Stage II patients, after initial adaptation, may reactivate psychological capital through meaning-seeking (e.g., reevaluating life values) and regaining control (109). However, late-stage patients (Stage III/IV) did not show significant associations, possibly due to the nonlinear depletion of psychological resources. When disease progression exceeds individual coping thresholds, psychological capital may collapse even with social support (110). Notably, Stage II patients had a 10.07-fold increased risk of belonging to the “moderate psychological capital-moderate death anxiety” group, suggesting that mid-disease psychological adaptation is a dynamic balancing process rather than a unidirectional improvement.

#### 4.2.5 Social support

According to the social support buffering theory, social support networks not only alleviate stress but also actively construct psychological resources. Instrumental support may enhance a sense of disease control, emotional support promotes positive emotions, and affiliational support (e.g., peer groups) reconstructs self-concept through social identification (111). Notably, the effect of social support on the “moderate psychological capital-moderate death anxiety” group weakened to an OR of 2.722, consistent with the law of diminishing marginal utility. When death anxiety reaches moderate levels, the transformation efficiency of the support system may decrease. This suggests that interventions should be designed in layers: for high-anxiety groups, structured support should be prioritized, while for moderate-anxiety groups, the quality of support networks should be optimized. Digital support tools may overcome the spatial and temporal limitations of traditional support, but their effectiveness requires further evidence-based validation.

This study revealed that social support is a core driver for enhancing psychological capital and alleviating death anxiety, with its effect exceeding the influence of demographic and clinical variables. This finding corroborates the universality of the social support buffering theory (112). Social support operates through its instrumental, emotional, and affiliational dimensions. Instrumental support directly reduces objective stress loads and enhances disease control (113); emotional support regulates anxiety responses by modulating neural circuits (114); and affiliational support reconstructs social identities, transforming patients into survivors and strengthening psychological resilience (115). However, in the “moderate psychological capital-moderate death anxiety” group, the effect size of social support weakened to OR=2.722 (95% CI=[1.893, 3.915], p < 0.001), aligning with the stress-support dynamic balance model (116). This suggests the need for layered intervention strategies: for high-anxiety groups, prioritize structured instrumental support, while for moderate-anxiety groups, focus on enhancing the quality of emotional support.

#### 4.2.6 Perceived stress

This study found that the individual perceived stress level had no significant predictive power for the latent profile of psychological capital and death anxiety. This seemingly counterintuitive result in fact profoundly reflects the psychological adaptation mechanism in the unique disease context of advanced pancreatic cancer. Pancreatic cancer itself constitutes an overwhelming and persistent threat to survival due to its high degree of malignancy, poor prognosis (117), and often accompanied by severe pain and digestive dysfunction. In this context, the patient’s cognitive assessment of the disease itself and the primary stress response triggered by it may become the influencing factors that dominate the patient’s psychological state (118). In contrast, the intensity and salience of general perceived stressors in daily life may be weakened or decentralized in the face of extreme survival crises. In other words, when individuals are exposed to the ultimate stress situation of advanced pancreatic cancer, their perception of stress is fundamentally reconstructed. The importance of daily stressors is relatively diminished, and the disease itself and the threats related to its existential meaning become the overwhelming cognitive focus. This cognitive reappraisal process may be the core psychological mechanism responsible for the insignificant effect of general perceived stress on deep psychological profiles. At the same time, individuals may develop specific psychological resilience or emotional numbness in the long-term process of coping with extreme disease stress (119), which alters their sensitivity to stress and further cushions the impact of perceived stress on deep psychological structures.

However, this result may also be limited by the study design and measurement tools. Factors such as the size of the sample, the selection criteria of the study subjects, and the sensitivity of the measurement instrument may affect the stability of the results. Future studies with larger sample sizes and more rigorous study designs are needed to verify the robustness of this finding. At the same time, it is necessary to further analyze whether perceived stress may act as a regulatory mechanism to affect the relationship between them, which will help to better understand the psychological adaptation process of pancreatic cancer patients. In clinical practice, while paying attention to the physiological symptoms of patients, clinical medical staff should pay attention to the assessment and intervention of psychological capital, improve the psychological resilience of patients through psychological support and psychological intervention, so as to improve their overall quality of life.

### 4.3 Practical implications

From a practical perspective, this study offers valuable guidance for psychological interventions in patients with pancreatic cancer. First, the results indicate that psychological capital and social support are key factors in alleviating death anxiety. Therefore, clinical psychological interventions should focus on enhancing psychological capital and building social support systems. Specifically, interventions such as psychological counseling, support groups, and family therapy can be employed to strengthen patients’ psychological capital and improve their social support networks. Particularly for patients in the “low psychological capital-high death anxiety” category, more personalized and intensified psychological support measures are recommended to alleviate their death anxiety and improve their quality of life.

This study also provides a stratified practical strategy for psychological interventions in pancreatic cancer patients. Through latent profile analysis, the study categorizes patients into three groups, each with distinct psychological characteristics and clinical needs. For example, patients in the “high psychological capital-low death anxiety” group may require less professional psychological intervention. In contrast, those in the “moderate psychological capital-moderate death anxiety” group may need stable psychological support and follow-up services. In contrast, patients in the “low psychological capital-high death anxiety” group should be prioritized for psychological interventions. This classification strategy can help clinicians more accurately identify high-risk patients and develop personalized psychological intervention plans tailored to their needs.

The data further show that each unit increase in social support significantly increases the probability of patients belonging to the “high psychological capital-low death anxiety” group. This suggests that healthcare institutions should actively help patients construct their social support networks, including family, friends, and community resources. Especially in rural areas with relatively scarce resources, healthcare institutions should strengthen collaboration with community resources to provide patients with more external support. Additionally, younger patients (18–45 years) may face higher life pressures and health risk perceptions and should receive special attention regarding their mental health needs.

The study found that females, urban residents, and Stage II patients exhibited significantly better psychological health, likely due to their greater access to high-quality medical resources and social support. Therefore, policymakers should focus on the mental health issues of rural residents and early-stage patients to ensure the equitable distribution of mental health resources. Furthermore, this study emphasizes the importance of interdisciplinary collaboration, suggesting the integration of psychology, sociology, and clinical medicine to establish a comprehensive system for managing mental health. Through cross-departmental collaboration, high-risk individuals can be screened more effectively, and timely psychological interventions can be provided to improve the overall survival quality and mental health of pancreatic cancer patients.

### 4.4 Limitations

Although this study incorporated randomization at the recruitment centers and random selection from lists of eligible patients at participating hospitals, it primarily employed convenience sampling. The data were exclusively collected from multiple tertiary grade A hospitals in Nanchong and Xi’an, which inherently limited the geographic, socioeconomic, and cultural diversity. For instance, the urban bias in the sample may have overestimated the protective effect of urban residency on psychological capital, as rural patients often face greater barriers, such as limited access to mental health services and stigmatization. Therefore, the generalizability of the findings is restricted and may not fully represent pancreatic cancer patients in either Chinese or international contexts, where regional disparities in healthcare resources could influence patients’ psychological states. Future studies should adopt multicenter, stratified random sampling across diverse settings, including rural and international locations, to enhance external validity and validate subgroup proportions.

The cross-sectional design further precluded causal inferences between variables, such as whether social support directly enhances psychological capital or whether bidirectional effects exist. The inability to track temporal dynamics further complicates this limitation. For example, the higher psychological capital observed in stage II patients may reflect post-diagnosis adaptation rather than a causal relationship, yet unmeasured variables, such as the timing of chemotherapy or surgery, were not controlled for, potentially leading to confounded results. Similarly, the single-time-point latent profile analysis classification cannot capture within-individual changes in subgroup membership. To address these limitations, future research is recommended to adopt longitudinal designs with repeated measurements and causal models, such as cross-lagged panel analysis or propensity score matching, which could integrate treatment timelines and thereby distinguish acute from chronic effects on death anxiety.

Moreover, this study did not comprehensively assess potential confounding factors, including cultural norms, religious beliefs, and work-family conflicts. These omissions introduced biased estimates in multivariate analyses, as single-variable results already indicated demographic influences, but without deeper exploration of mediating pathways. The lack of biomarkers or physiological indicators further reduced the ability to link psychosocial findings to potential disease mechanisms, such as how heightened death anxiety might accelerate pancreatic cancer progression through neuroendocrine dysregulation. Future studies should employ structural equation modeling to test mediating/moderating factors, use mixed methods for qualitative analyses of cultural/familial factors, and integrate multi-omics data with longitudinal tracking to develop dynamic biopsychosocial predictive models. These approaches not only enhance subgroup classification but also provide precise interventions based on patients’ evolving needs.

## 5 Conclusion

This study reveals the heterogeneous interplay between psychological capital and death anxiety among pancreatic cancer patients through latent profile analysis, identifying three distinct subgroups characterized by varying levels of psychological resilience and existential distress. The significant negative correlation between psychological capital and death anxiety, coupled with the protective role of social support, underscores the critical importance of psychosocial resources in mitigating emotional burden. Notably, demographic disparities—such as younger age, male gender, rural residency, and advanced cancer stages—highlight the need for culturally sensitive interventions tailored to vulnerable populations. While the findings advance theoretical frameworks by integrating dynamic interactions between psychological states and socio-environmental factors, limitations in sample diversity and cross-sectional design necessitate longitudinal and multicentric validation. Future research should prioritize biopsychosocial models that link psychological interventions (e.g., narrative therapy for death anxiety) with biomarkers of stress and immune function, ultimately fostering precision medicine strategies to enhance holistic care for pancreatic cancer patients.

## Data Availability

The original contributions presented in the study are included in the article/**Supplementary Material**. Further inquiries can be directed to the corresponding authors.
