# Supplementary material for: Psychological capital and death anxiety in pancreatic cancer patients: a latent profile analysis
Source: Front Psychiatry. 2025 Sep 10;16:1627422. doi: 10.3389/fpsyt.2025.1627422 (PMC12457670; doi:10.3389/fpsyt.2025.1627422)
Supplement: Supplementary file 1 [file Supplementaryfile1.docx]

Appendix A

| Variables | Measurement items | Scale source |
| --- | --- | --- |
| Psychological Capital | Do many people appreciate my talents? | Zhang (2010) |
|  | Am I a person who doesn't get angry easily? |  |
|  | Do my insights and abilities exceed those of the average person? |  |
|  | When encountering setbacks, can I recover quickly? |  |
|  | Do I have great confidence in my own abilities? |  |
|  | Do I rarely care about the unpleasant things in life? |  |
|  | Can I always complete tasks excellently? |  |
|  | Will a bad experience make me feel depressed for a long time? |  |
|  | When facing difficulties, will I calmly seek solutions? |  |
|  | Do I feel that my life is very tiring? |  |
|  | Am I willing to take on difficult and challenging work? |  |
|  | When things don't go my way, do I easily become dejected? |  |
|  | When in an adverse situation, will I actively try different strategies? |  |
|  | When under a lot of pressure, will I have a poor appetite and trouble sleeping? |  |
|  | Will I actively study or work to achieve my ideals? |  |
|  | When the situation is uncertain, do I always expect good results? |  |
|  | Am I working hard to achieve my goals? |  |
|  | Do I always see the good side of things? |  |
|  | Will I pursue my goals with full confidence? |  |
|  | Do I think that good people are still in the majority in society? |  |
|  | Do I have a certain plan for my own study and life? |  |
|  | Most of the time, am I in high spirits? |  |
|  | Am I very clear about the kind of life I want? |  |
|  | Do I think that life is beautiful? |  |
|  | Do I know what the goal of my life is? |  |
|  | Do I think that the future is full of hope? |  |
| Perceived Social Support | Is there a special person who is around when I am in need? | Zimet et al. (1988) |
|  | Is there a special person with whom I can share my joys and sorrows? |  |
|  | Does my family really try to help me? |  |
|  | Do I get the emotional help and support I need from my family? |  |
|  | Do I have a special person who is a real source of comfort to me? |  |
|  | Do my friends really try to help me? |  |
|  | Can I count on my friends when things go wrong? |  |
|  | Can I talk about my problems with my family? |  |
|  | Do I have friends with whom I can share my joys and sorrows? |  |
|  | Is there a special person in my life who cares about my feelings? |  |
|  | Is my family willing to help me make decisions? |  |
|  | Can I talk about my problems with my friends? |  |
| Death Anxiety | Am I very much afraid to die? | Templer (1970) |
|  | Does the thought of death seldom enter my mind? |  |
|  | Doesn't it make me nervous when people talk about death? |  |
|  | Do I dread to think about having to have an operation? |  |
|  | Am I not at all afraid to die? |  |
|  | Am I not particularly afraid of getting cancer? |  |
|  | Does the thought of death never bother me? |  |
|  | Am I often distressed by the way time flies so very rapidly? |  |
|  | Do I fear dying a painful death? |  |
|  | Does the subject of life after death trouble me greatly? |  |
|  | Am I really scared of having a heart attack? |  |
|  | Do I often think about how short life really is? |  |
|  | Do I shudder when I hear people talking about a World War III? |  |
|  | Is the sight of a dead body horrifying to me? |  |
|  | Do I feel that the future holds nothing for me to fear? |  |
| Perceived Stress | In the last month, how often have you been upset because of something that happened unexpectedly? | Cohen et al. (1983) |
|  | In the last month, how often have you felt that you were unable to control the important things in your life? |  |
|  | In the last month, how often have you felt nervous and "stressed"? |  |
|  | In the last month, how often have you dealt successfully with irritating life hassles? |  |
|  | In the last month, how often have you felt that you were effectively coping with important changes that were occurring in your life? |  |
|  | In the last month, how often have you felt confident about your ability to handle your personal problems? |  |
|  | In the last month, how often have you felt that things were going your way? |  |
|  | In the last month, how often have you found that you could not cope with all the things that you had to do? |  |
|  | In the last month, how often have you been able to control irritations in your life? |  |
|  | In the last month, how often have you felt that you were on top of things? |  |
|  | In the last month, how often have you been angered because of things that happened that were outside of your control? |  |
|  | In the last month, how often have you found yourself thinking about things that you have to accomplish? |  |
|  | In the last month, how often have you been able to control the way you spend your time? |  |
|  | In the last month, how often have you felt difficulties were piling up so high that you could not overcome them? |  |

**References**

Cohen, S., Kamarck, T., & Mermelstein, R. (1983). A global measure of perceived stress. *J Health Soc Behav*, *24*(4), 385-396.

Templer, D. I. (1970). The construction and validation of a Death Anxiety Scale. *J Gen Psychol*, *82*(2d Half), 165-177. <https://doi.org/10.1080/00221309.1970.9920634>

Zhang, K. (2010). Positive psychological capital: measurement and relationship with mental health. *Studies of Psychology and Behavior*, *8*, 58.

Zimet, G. D., Dahlem, N. W., Zimet, S. G., & Farley, G. K. (1988). The multidimensional scale of perceived social support. *Journal of personality assessment*, *52*(1), 30-41.
